# Predictors of health-related quality of life after total knee arthroplasty: a case–control study

**DOI:** 10.1038/s41598-024-65042-z

**Published:** 2024-06-19

**Authors:** Alireza Askari, Mehdi Mohammadpour, Mahmoud Jabalameli, Niloofar Naeimipoor, Babak Goodarzy, Behnam Jafari, Heeva Rashidi, Fatemeh Mousazadeh, Maziar Rajei, Amir Khazanchin, Mansour Bahardoust, Mohammad Hassanzadeh

**Affiliations:** 1 Department of Orthopedic Surgery, School of Medicine, Iran University of Medical Sciences, Tehran, Iran; 2 Bone and Joint Reconstruction Research Center, Shafa Orthopedic Hospital, Iran University of Medical Sciences, Tehran, Iran; 3 Clinical Research Development Unit, Taleghani Educational Hospital, Abadan University of Medical Sciences, Abadan, Iran; 4 Department of Psychology, Neyshabur Branch, Azad University, Neyshabur, Iran; 5 School of Medicine, Iran University of Medical Sciences, Tehran, Iran; 6 Department of Epidemiology, School of Public Health, Shahid Beheshti University of Medical Sciences, Tehran, Iran

**Keywords:** Total knee arthroplasty, Health, Related quality of life, 36-item short-form health survey, Iranian population, Ageing, Bone, Diseases

## Abstract

Total knee arthroplasty (TKA) improves patients’ Health-related quality of life (HRQoL) compared to before surgery. However, based on our knowledge, the improvement in HRQoL after TKA, which depends on various factors, has yet to be investigated compared to healthy people. This study aimed to evaluate the HRQoL of patients compared to healthy people and the factors affecting the HRQoL after TKA. In this matched case–control study (1002 participants), HRQoL in 501 patients who underwent TKA between 2015 and 2022 at Shafa Yahyainan Hospital affiliated with Iran University of Medical Sciences were compared with 501 healthy controls. HRQoL was evaluated in two parts (before compared to 12 months after TKA and 12 months after TKA compared to the healthy population). The 36-item short-form health survey (SF-36) was used to evaluate HRQoL 12 months after surgery. The influencing factors on HRQoL were evaluated by multivariate logistic regression analysis. No significant difference was observed in the demographic characteristics of the participants in the two groups. The mean overall SF-36 score, 12 months after surgery, significantly improved compared to before surgery (64.21 ± 22.2 vs. 37.55 ± 15.13, *p*:0.001). The mean total score of SF-36 was statistically similar between the case and control groups (64.21 ± 22.2 VS 72.53 ± 25.3). The multivariate analysis showed that sex, BMI, number of comorbidities, postoperative compliance, and complications were significantly related to the decrease in patients’ HRQoL (*P* < 0.001).TKA can improve the HRQoL except for two subscales of happiness/vitality and physical performance, similar to the healthy population. Female gender, obesity and overweight, comorbidity, bilateral TKA, non-adherence to postoperative physiotherapy, and complications were associated with decreased HRQoL.

## Introduction

Knee osteoarthritis (OA) is one of the most common joint diseases in the world, which causes significant disabilities, especially in the elderly^1–3^. This disease leads to the involvement of both knees and the pain and functional disorder caused by this, often with the patient’s inability to ambulate and walk in advanced stages, disrupts the patients’ Health-related quality of life (HRQoL)^4,5^. Total knee arthroplasty (TKA) is one of the most common joint replacement procedures worldwide, known as the end stage of treatment for these patients^5–7^. Due to the aging population and the increase in life expectancy, TKA is increasing worldwide^8^. This amount is estimated to increase by 637% (3.48 million) from 2005 to 2030 in the United States of America^9^.

Although total Joint Arthroplasty (TJA) can significantly improve pain, mobility, and physical function, not all patients are satisfied with the outcome^10,11^. Postoperative complications such as thromboembolism, neurovascular injuries, prosthesis instability, prosthesis fracture, limb length discrepancies, and revision have been associated with TKA, leading to a reduction in patients’ quality of life. Among the reported complications, wound complications often lead to reoperation or revision^12–15^. As the number of people with knee prostheses continues to increase, there is a growing interest in identifying predictors of quality of life after TKA^15–17^. Therefore, accurate assessment of functional outcomes and HRQoL of patients after TKA is essential^18^.

Based on studies conducted in different countries, patients’ quality of life after TKA depends on various factors such as age, sex, comorbidities, race, body mass index, etc.^19–21^. Evaluation of health-related quality of life (HRQoL) is a scale used to evaluate the success of TKA, which can evaluate the HRQoL of patients in different dimensions. Pain and improvement in the performance and function of the patient are strong predictive factors in HRQoL after TKA^22,23^. Silva et al. showed that TKA improved patients’ quality of life, especially six months after TKA^5^.

Here, we aim to evaluate the HRQoL of post-TKA patients compared to the healthy population and investigate factors that may play a predictive role in this scenario. It is assumed that with the similarity of other demographic characteristics and underlying diseases in the two groups, TKA can bring the HRQoL of patients with OA closer to the normal population. According to our knowledge, no comprehensive study of the HRQoL and factors affecting the HRQoL after TKA has been investigated in the Iranian population. Therefore, considering the importance of this issue, this study aims to investigate the HRQoL of patients with TKA in eight dimensions and the factors affecting the HRQoL after TKA.

## Materials and methods

### Patients, design, and setting

This study was approved by the ethics committee of Iran University of Medical Sciences with code IR.IUMS.FMD.REC.1402.141. All research was performed by relevant guidelines/regulations, and informed consent was obtained from all participants and/or their legal guardians. In this case–control study, 501 patients with OA of the knee who underwent TKA in Shafa Yahyayan Hospital between 2015 and 2022 were compared with 501 healthy controls. To control for confounding variables, cases and controls were matched by frequency matching regarding age, gender, body mass index, comorbidity, smoking, alcohol consumption, and education level. The control group was selected from among healthy people referring to the hospital who had no history of knee joint involvement and TKA. The sampling method of patients was done as available and among the patients who met the criteria for inclusion in the study. Informed consent was obtained from all patients.

### Inclusion and exclusion criteria

Inclusion criteria included: history of TKA, aliveness of the patient at the time of follow-up, patients who had at least 12 months of surgery, patients who had a file, and cooperation for follow-up and completing the questionnaire. History of previous knee surgery for any reason, secondary TK or revision; Total hip arthroplasty(THA), cancer in any organ, drug, and alcohol addiction; patients with untreated severe depression or other neuropsychiatric diseases, including MS, Parkinson’s, and Alzheimer’s, and patients with chronic viral infection including Viral hepatitis and HIV, and death were defined as exclusion criteria.

### Data collection

Demographic data of cases and controls (age, gender, body mass index (BMI), education level, comorbidities, smoking, alcohol consumption, follow-up, and DJD grade) was collected using a checklist. Clinical findings, revision, and operation complications were also evaluated for the patients. To examine the HRQoL, the SF-36 questionnaire was used, and the validity and reliability of which Montazeri et al. confirmed in Iran^24^. This questionnaire contains 36 questions in eight dimensions (physical performance, physical role, physical pain, general health, vitality, social performance, emotional role, and mental health).

The study’s objectives were evaluated in two parts: 1. Comparing the quality of life of patients before surgery and 12 months after TKA; 2. Comparing the quality of life of patients 12 months after TKA compared to the healthy population. After obtaining consent from the patients to participate in the study, the HRQoL questionnaire was completed by the patients or by the researcher (in cases where the patient could not complete the questionnaire) 12 months after TKA.

The scores obtained in eight separate areas were then calculated as a total score, and all areas were adjusted and reported separately based on one hundred. The mean was between 0 and 100. A higher score means a better HRQoL. PCS and MCS out of 100 were calculated and recorded. PCS and MCS defined two aspects (physical and mental) of HRQoL^25^.

### Sample size collection

The appropriate sample size for conducting the study, according to the effect size estimate of 0.42 for the difference in the overall mean SF-36 score in patients undergoing TKA based on the study of I Papakostidou et al.^26^ with an α error of 5% and a power of 90% by the epidemiologist using G power version 3.1 (Erdfelder, Faul, & Buchner, 1996) software, 411 people was estimated for each group.

### Statistical analysis

The data was analyzed using SPSS version 22 statistical software (IBM. -SPSS, New York, USA). Descriptive statistics (frequency and %) were used to report qualitative variables. Quantitative variables were reported using mean and standard deviation. The normal distribution of quantitative variables, such as the mean score of the questionnaire, was evaluated using the Shapiro–Wilk test. The chi-square test was used to compare qualitative variables in two groups. A paired samples *t*-test was used to compare quantitative variables in matched case–control groups, assuming a normal distribution of quantitative variables. If the assumption of normality was not established, the Wilcoxon test was used. A paired samples *t*-test was used to compare the mean HRQoL before and 12 months after surgery. All variables with a *P* less than 0.15 in the univariate analysis were entered into multivariate Conditional logistic regression to control the confounding variables. The effect size was reported with the adjusted odds ratio (OR) in the 95% confidence interval (95% CI). Conditional logistic regression analysis was used to estimate the variables predicting the quality of life in TKA patients. A level of statistical significance of less than 0.05 was considered.

## Results

Overall, 501 cases and 501 controls completed the study. The mean age of the case and control groups was 66.74 ± 7.71 and 66.89 ± 6.95 years, respectively. (*p*:0.89) 429(85.6%) of case group and 422(84.2%) controls were women. The mean follow-up of the patients was 25.8 ± 3.65 months. No significant difference was observed in demographic characteristics between the two groups. (*P* > 0.05) Table 1.

**Table 1:** Comparison of demographic characteristics in two groups.

Variable	Group		*P* value
	Case (N = 501)	Control (N = 501)	
Age (year)	66.74 ± 7.71	66.89 ± 6.95	0.89
*Gender*			
Female Male	429 (85.6%) 72(14.4%)	422 (84.2%) 79 (15.8)	0.71
BMI (kg/m^2^)	29.66 ± 4.58	30.1 ± 4.68	0.35
*Educational status*			
Illiterate < diploma Diploma > diploma	52 (10.4%) 126 (25.2%) 135 (26.9%) 188 (37.5%)	47 (9.3%) 132 (26.4%) 126 (25.2%) 196 (39.1%)	0.26
*Smoker*			
Positive Negative	188 (37.5%) 313 (62.5%)	192 (38.3%) 309 (61.7%)	0.42
*Number of comorbidities*			
0 1 2 > 2	252 (50.3%) 189 (37.7%) 39 (7.8%) 21 (4.2%)	245 (48.9%) 192 (38.3%) 44 (8.8%) 20 (4%)	0.11

### Complication 12 months after TKA

In general, 15 (3%) had complications 12 months after TKA, which included seven (1.4%) periprosthetic joint infection (PJI)), 2 Aseptic loosening, 1 DVT, one periprosthetic fracture, one fracture, and 3 MCL instability.

### HRQoL 12 months after TKA

The mean overall SF-36 score 12 months after surgery significantly improved compared to before surgery (64.21 ± 22.2 vs. 37.55 ± 15.13, *p* < 0.001). Also, the mean score in all SF-36 subscales improved significantly twelve months after TKA compared to before surgery. (*p* < 0.05) Table 2.

**Table 2:** Comparison of the SF-36 questionnaire total and sub-scales’ score in cases before and 12 month after TKA.

SF-36 sub-scale	501 patients underwent TKA		*P* value
	Before TKA	12 month after TKA	
Physical performance	32.2 ± 19.3	65.2 ± 25.3	< 0.001
Physical role	43.2 ± 14.2	75.7 ± 21.2	< 0.001
Mental health	50.23 ± 12.33	69.38 ± 22.2	< 0.001
Happiness and vitality	24.25 ± 17.16	50.5 ± 20.16	< 0.001
Emotional role	35.33 ± 15.6	62.2 ± 25.2	< 0.001
Social role	31.2 ± 19.2	55.5 ± 21.6	< 0.001
Physical pain	24.41 ± 12.3	55.68 ± 32.5	< 0.001
General health	50.11 ± 18.3	79.25 ± 21.2	< 0.001
Total score	37.55 ± 15.13	64.21 ± 22.2	< 0.001
MCS	38.25 ± 9.54	50.89 ± 9.2	< 0.001
PCS	25.33 ± 7.33	42.58 ± 9.2	< 0.001

Twelve months after surgery, although the mean overall SF-36 score in the case group (64.21 ± 22.2) was slightly lower than the control group (72.53 ± 25.3), this difference was not statistically significant. (*P*: 0.061) While in two sub-scales, including Happiness/vitality and Physical performance, the mean score in the case group was still lower than the control (*p* < 0.05). The mean PCS score in the case group was significantly lower than the control group (42.58 ± 9.2 Vs. 61.33 ± 8.5, *p*:0.001). No significant difference was observed in the MCS score in the two groups (*P*: 0.62) Table 3.

**Table 3:** Comparison of the SF-36 questionnaire total and sub-scales’ score in case and controls.

Sub-scale	Group		*P* value
	Case (N = 501)	Control (N = 501)	
Physical performance	65.2 ± 25.3	76.78 ± 22.1	0.006
Physical role	75.7 ± 21.2	83.25 ± 12.2	0.098
Mental health	69.38 ± 22.2	71.25 ± 25.5	0.11
Happiness and vitality	50.5 ± 20.16	75.66 ± 17.1	0.001
Emotional role	62.2 ± 25.2	65.2 ± 25.2	0.39
Social role	55.5 ± 21.6	58.25 ± 25.5	0.16
Physical pain	55.68 ± 32.5	63.25 ± 33.2	0.086
General health	79.25 ± 21.2	86.2 ± 11.2	0.62
Total score	64.21 ± 22.2	72.53 ± 25.3	0.061
MCS	50.89 ± 9.2	52.35 ± 8.65	0.62
PCS	42.58 ± 9.2	61.33 ± 8.5	0.001

*p* value less than 0.05 is considered significant.

### Factors associated with HRQoL after TKA

The results of the univariate analysis showed that age, sex, BMI, Educational Level, Number of comorbidities, and Complications were significantly associated with the HRQoL of patients. (*p* < 0.05) Table 4. The results of multivariate analysis showed that female gender, BMI > 30(kg/m2), number of comorbidities > 2, bilateral TKA, non-compliance, and complications were significantly related to the decrease in patients’ HRQoL. (*P* < 0.001) Table 5.

**Table 4:** The results of univariate analysis.

Variable	501 patients underwent TKA	
	Mean total SF-36	*P* value
*Age (year)*		
< 40 40–60 > 60	67.24 ± 19.25 66.58 ± 20.33 59.25 ± 18.66	0.048
*Gender*		
Female Male	58.12 ± 17.25 70.25 ± 12.33	0.035
*BMI (kg/m*^*2*^*)*		
20–25 25.1–30 > 30	78.24 ± 12.63 63.99 ± 11.58 52.33 ± 20.36	0.001
*Educational level*		
< diploma ≥ diploma	59.19 ± 20.35 74.36 ± 16.31	0.023
*Smoker*		
Positive Negative	62.55 ± 21.56 65.61 ± 20.54	0.17
*Number of comorbidities*		
< 2 > 2	60.65 ± 19.8 73.51 ± 20.5	0.036
*Complication*		
No Yes	62.35 ± 20.32 80.25 ± 12.52	0.001
*Compliance with physiotherapy*		
No Yes	59.34 ± 18.6 78.25 ± 19.1	0.001
*TKA side*		
Uni lateral Bilateral	80.22 ± 18.55 54.22 ± 22.3	0.001

**Table 5:** Factors related to quality of life based on the results of multivariate regression analysis.

Variable	OR adj	95% CI		*P* value
		Lower	Upper	
Sex (Female Vs Male)	1.78	1.09	2.48	0.025
BMI > 30 Vs < 30 (kg/m2)	2.54	1.25	3.84	0.001
Number of comorbidities > 2 Vs < 2	1.45	1.02	1.83	0.04
Complications yes Vs. No	2.09	1.13	3.05	0.001
compliance with physiotherapy (No Vs. Yes)	1.92	1.09	2.71	0.012
Bilateral vs. unilateral	1.88	1.08	2.69	0.033

## Discussion

Recently, studies have shown that HRQoL improves significantly after TKA. However, very limited studies have compared the extent of this improvement with a control group. Postoperative recovery varies among countries, populations, and even regions of the same country due to differences in access to postoperative care and social and economic conditions^5,18,27,28^. Unlike THA, where the functional outcome and HRQoL in patients are somewhat clear, the HRQoL of patients after TKA compared to healthy people and the factors affecting it are still unclear for orthopedic surgeons^29–31^. Therefore, considering the importance of this issue, the aim of this matched case–control study, for the first time in Iran, was to evaluate the HRQoL of patients 12 months after TKA and the factors affecting it on 1002 participants.

Our study showed that 12 after TKA, the mean overall score and all subscales of SF-36 were significantly higher than before surgery. Compared to the healthy population, the mean overall SF-36 score was slightly lower in the case group than in the control group. However, this difference was not statistically significant, and the mean overall SF-36 score of the patients 12 months after TKA was similar to the healthy population. The comparison of the subscales showed that, apart from the two scales of happiness and vitality and physical performance, whose mean score in the case group was lower than the control, no significant difference was observed in the other subscales for the mean score in the two groups. The mean pain subscale score was similar in both groups. No significant difference was observed for the mean score of MCS in the two groups. The average score of PCS in the control group was higher than the case group, which can be due to the lower mean score of the subscales of physical performance and physical role in the case group compared to the control group, which was in line with the studies done in this field^5,27,32,33^. Also, in our study, the rate of PJI after TK was 1.4%, consistent with the results of studies that reported the rate of septic after primary TK between 1 and 5%^34,35^.

A Neuprez et al., in 2020, showed that the overall mean SF-36 score improved significantly one year after the TKA, which confirms the results of our study. They showed that the highest change in the mean score of SF36 subscales occurred in the first year after TKA. Pain is significantly reduced in the first months after TKA, which improves patients’ HRQoL^28^. In a systematic review, RR Silva et al., showed that despite the continued improvement in the HRQoL after TKA, the physical subscale may still be lower than that of healthy people. Better dynamic balance, less lameness, better sleep quality, less pain, physical activity, adequate social and family support, and meeting patients’ expectations after TKA surgery improve patients’ quality of life after TKA^5^. In our study, one year after the TKA, the happiness /vitality and physical performance were lower in the case group than in the healthy subjects, which indicates that it takes more time to return to activity and perform physical activities compared to other dimensions. In line with the results of our study, in a meta-analysis, S Witjes et al., reported that the return to sport after TKA is very wide and varies in the range of 36% to 89%. They showed that only 36% of patients returned to the same level of exercise. The rate of return to physical activities is different based on unilateral or bilateral arthroplasty, and this rate was higher and in the range (of 74–100%) in people who underwent unilateral knee arthroplasty surgery^36^. In our study, patients’ quality of life and physical performance were higher in patients who underwent unilateral arthroplasty than bilateral arthroplasty. The better quality of life in patients who underwent unilateral TKA can be justified because OA may have been diagnosed earlier in these patients, and they underwent TKA earlier. In a systematic review, L Shan et al.^27^ showed that TKA is associated with improving the HRQoL of patients, especially the dimensions of pain and function, which leads to positive patient satisfaction. In another study, CA Jones et al.^37^ showed that TKA significantly improved HRQoL after TKA. In 2021, Y Al Thaher et al.^38^ showed that TKA significantly improved all dimensions of HRQoL compared to before surgery. O Bruyère et al. showed Six months after TKA, significant improvement was observed in two of the eight SF-36 dimensions (i.e., physical function and pain) compared to preoperative, which confirms the results of our study^31^.

In addition, our study showed, female gender, BMI > 30(kg/m2), the occurrence of complications after TKA, comorbidity > 2, and non-compliance with physiotherapy after TKA were significantly related to the decrease in quality of life of patients after TKA, which was consistent with the results of studies conducted in this field^5,39,40^.

In a systematic review study, RR Silva et al., showed that obesity and comorbidities were significantly associated with decreased patients’ HRQoL^5^. E Yakobov et al. showed the female gender and BMI > 30(kg/m2) were significantly associated with a decrease in patients’ HRQoL after TKA^41^. M Núñez et al., showed that obesity and complications after TKA were significantly associated with a decrease in the HRQoL of patients^42^. Xu et al. showed that the HRQoL in obese and non-obese patients significantly improved after TKA. The improvement rate was higher in patients with normal BMI^39^. These results confirmed the results of our study. In our study, BMI > 30 kg/m2 was significantly related to the decrease in HRQoL of patients. A high BMI prevents a quick return to activity and can be associated with more pain during activity due to the higher weight.

Similar to the results of our study, DL Snell et al., showed that comorbidity was significantly related to the reduction of patients’ HRQoL in TKA patients^43^. Concurrently suffering from other comorbidities delays patients’ recovery and return to physical activities. In addition, comorbidity with an increased risk of postoperative complications can lead to decreased HRQoL in patients after TKA. The occurrence of complications increases the burden of the disease by increasing direct and indirect costs, and in some cases, the patient needs revision, which leads to a decrease in the quality of life of patients after TKA.

JFM Verbeek et al., showed that the female gender was associated with decreased quality of life after TKA^44^. In our study, the HRQoL was higher in men than in women, which the lower tolerance level of women can explain compared to men. Contrary to the results of our study, CA Jones et al.^45^ did not show a significant relationship between the gender of the patients and the quality of life of the patients after TKA, which could be due to the difference in the demographic and social characteristics of the patients. The follow-up period and sample size examined in the two studies can be justified.

Our study showed that physical therapy after TKA can be related to improving the HRQoL of patients. In line with the results of our study, N Büker et al., showed that physical therapy after TKA was associated with improved functional outcomes and HRQoL of patients^46^. In another study, M Bahardoust et al., showed in a case–control study that post-operative care and adherence to physiotherapy were associated with improved HRQoL of patients after THA^29^. Physiotherapy can help the patient return to daily activities faster because during the period of OA and before surgery, the patient’s muscles are significantly weakened due to lack of use, and physiotherapy can be associated with better results by strengthening the muscles. Because the studies that examined the HRQoL of patients after TKA with a healthy group were very limited, we could not compare the results of our study with studies with similar and opposite results compared to a healthy group.

There were strengths and weaknesses in our study that should be pointed out. In this study, we could not estimate several variables, including the duration of OA before TKA, which affect the study’s results. Also, this study was conducted on the population of Iranian OA patients. HRQoL can be different in races, countries, and even regions of a country depending on the availability of health care and post-operative care, and its results need to be more generalizable for other races. These results are not generalizable to other races, which was the most important limitation of this study. The most important strength of this study was the design of a matched case–control study with a high sample size to estimate HRQoL and factors affecting it for the first time in Iran.

## Conclusion

Our study showed that TKA can significantly improve the HRQoL of patients compared to before surgery. Twelve months after TKA, the mean overall SF-36 score in patients who underwent TKA was similar to the healthy population except for the two subscales of happiness/vitality and physical performance. Female gender, obesity, overweight, comorbidity, bilateral TKA, non-compliance with postoperative physiotherapy, and complications were related to the decrease in HRQoL.

## Data Availability

The datasets used during the current study available from the corresponding author on reasonable request.
